# Protective effect of vitamin E on sperm parameters, chromatin quality, and DNA fragmentation in mice treated with different doses of ethanol: An experimental study

**DOI:** 10.18502/ijrm.v19i6.9374

**Published:** 2021-07-27

**Authors:** Mohamad Reza Doostabadi, Mohammadmehdi Hassanzadeh-taheri, Mahmoud Asgharzadeh, Masoomeh Mohammadzadeh

**Affiliations:** ^1^Department of Reproductive Biology, Research and Clinical Center for Infertility, Yazd Reproductive Sciences Institute, Shahid Sadoughi University of Medical Sciences, Yazd, Iran.; ^2^Department of Anatomy, Faculty of Medicine, Birjand University of Medical Sciences, Birjand, Iran.; ^3^Royesh Infertility Center, Birjand University of Medical Science, Birjand, Iran.; ^4^Birjand Cellular and Molecular Research Center, University of Medical Sciences, Birjand, Iran.

**Keywords:** Ethanol, Sperm parameters, Vitamin E.

## Abstract

**Background:**

Excessive consumption of alcohol induces an increase in oxidative stress production and can lead to detrimental effects on the male reproductive system.

**Objective:**

To evaluate the possible protective effects of coadministration of vitamin (vit) E on the detrimental changes in the sperm quality of mice administered ethanol.

**Materials and Methods:**

Fifty-four BALB/c mice were categorized into nine groups (n = 6/each). The control group received a basal diet while the eight experimental groups received ethanol 10%; ethanol 20%; vit. E 100 mg; vit. E 200 mg; ethanol 10% + vit. E 100 mg; ethanol 10% + vit. E 200 mg; ethanol 20% + vit. E 100 mg; ethanol 20% + vit. E 200 mg. After 35 days, the sperm parameters and sperm chromatin were assessed.

**Results:**

The results demonstrated a significant reduction in the motility rate, normal morphology rate, viability rate, increase in abnormal DNA structure and packaging (TB staining), and DNA damage (TUNEL) in ethanol consumer groups. In addition, the findings showed a significant increase in the aforementioned parameters in ethanol- and vit. E-consumer groups compared to the ethanol-only consumer groups. The ethanol group received 20% of the most damage among the groups. The group receiving vit. E 100 mg and those receiving ethanol 10% + vit. E 200 mg gained the highest benefit among the groups.

**Conclusion:**

Sperm forward progressive motility, normal morphology rate, and viability decreased in the ethanol groups. Also, the rates of spermatozoa with abnormal DNA structure and DNA fragmentation increased in the ethanol groups. Our findings revealed that the coadministration of vit. E and ethanol can protect destructive changes in DNA structure and damage.

## 1. Introduction

The American Society of Reproductive Medicine defines infertility as failure to achieve pregnancy after 1 yr timed unprotected intercourse (1). Approximately 15% of couples in the world suffer from infertility and 50% of cases are related to male causes (2). Many factors cause infertility, among them, ethanol, as a widely used drug, is well-known for suppressing reproductive function. Excessive consumption of ethanol is a significant public health problem.

Excessive consumption of alcohol results in a variety of pathological changes in male reproduction such as low sperm count, reduced motility and quality of spermatogenesis, altered testicular histology, and changes in sperm morphology such as breakage of the sperm head, distention of the mid-section, and tail curling in men and experimental animals (3). It has been suggested that the mechanisms associated with acute and chronic alcohol consumption and decreased sperm quality are related to the production of reactive oxygen species (ROS) and reduction of nicotinamide adenine dinucleotide, which enhances the activity of the respiratory chain, ROS formation, and It also affects the metabolism of male hormones such as testosterone and subsequently has an adverse effect on the process of spermatogenesis (4). In addition, one of the products produced in the process of alcohol metabolism is acetaldehyde, interacts with proteins and lipids to produces ROS (5) ROS targets and damages plasma membranes and DNA molecules in sperm and other cells (6). High levels of ROS such as superoxide ions, hydrogen peroxide, peroxynitrates cause damage to cell components such as membrane lipids, proteins, organelles, and cell DNA. In addition, Damage caused by oxidative stress can also affect gene expression (7, 8). A cohort study examining the effect of alcohol consumption in infertile individuals showed that alcohol consumption for 4–7 wk has no negative effect on sperm parameters (9).

“Antioxidants are the molecules that reduce the free radicals decreasing OS, for example, vitamin (vit.) A/C/E, glutathione, n-acetyl-l cysteine, uric acid, bilirubin, albumin, thiols, ubiquinol, bioflavonoids, carotenoids, etc.” (10). Vit. E (α-tocopherol) is a fat-soluble organic material and the primary antioxidant component of the spermatozoa that is beneficial for the maintenance of mammalian spermatogenesis (11). “Vit. E in the form of α-tocopherol, it is a major antioxidant located within biological membranes that play a role in protecting from lipid peroxidation. α-tocopherol breaks the chain reactions of lipid peroxidation through the mechanism of donation of a hydrogen atom from its phenolic hydroxyl group to lipid peroxyl radical resulting in the formation of stable lipid hydroperoxide and unreactive tocopheroxyl radicals” (10). This vitamin has strong antioxidant properties, inhibits lipid peroxidation, and is a protector against the ROS in the testes. A deficiency of vit. E leads to increased OS levels which are incompatible with normal spermatogenesis and testosterone production (12).

These observations strongly suggest that vit. E Maintains fertility of male by reducing oxidative stress. However, so far, the capacity of vit. E to prevent ethanol-induced toxicity in the reproductive system has not been determined. The beneficial effects were evident in the form of increase in testes weight, semen-quality parameters, antioxidants status, and testosterone in mammals as well as birds (13). Vitamin E increases the level of antioxidants in the body and reduces the effect of oxidative damage on the testicles and increases sperm motility (14).

Hence, this study was designed to investigate the probable protective effects of coadministration of vit. E and different doses of ethanol on the semen parameters and sperm DNA integrity of BALB/c mice.

## 2. Materials and Methods

### Animals and experimental design

In this experimental study, 54 healthy BALB/c mice (8-wk old, weighing 25 ± 2 gr) were purchased from the animal facility of the research center of experiments at the Medical University of Birjand, Iran. The mice were housed in clean polyethylene cages in standard condition, temperature-controlled room (22 ± 2°C), proper humidity (50 ± 5%) with a 12-hr light/dark cycle, and libitum access to water and food (Behparvar co., Iran). Then, they were randomly divided into nine groups (n = 6/each) as follow:

Group 1: The control group received a basal diet;

Group 2: Gavaged with 10% (V/V) ethanol (99% v/v, Merk, Germany) daily, respectively;

Groups 3 and 4: Gavaged with 10% (V/V) ethanol and injected with Vit. E (Osveh Co., Iran) 100, 200 mg/kg intraperitoneally, respectively;

Group 5: Gavaged with 20% (V/V) ethanol (99% v/v, Merk, Germany) daily, respectively;

Groups 6 and 7: Gavaged with 20% (V/V) ethanol and injected with Vit. E 100, 200 mg/kg intraperitoneally, respectively;

Groups 8 and 9: Received Vit. E 100, 200 mg/kg intraperitoneally, respectively.

### Epididymal sperm preparation

After 35 days, all mice underwent surgery and small pieces of epididymis were dissected and transferred into a 1-mL pre-warmed Ham's F10 medium (37°C, 5% CO${}_{2}$). The epididymal tissue was then gently cut with a needle to allow the sperm to swim into the medium and then placed in the incubator for 15 min.

### Sperm analysis

All protocols were done according to the National Institute of Health Guide for the care and use of laboratory animals.

#### Sperm motility

Motility was expressed as the percentage of progressive and nonprogressive spermatozoa. Progressive motility (PR): spermatozoa moving actively, either linearly or in a large circle, regardless of speed. Nonprogressive motility (NP): all other patterns of motility with an absence of progression, e.g., (15).

#### Sperm viability

Sperm vitality, as estimated by assessing the membrane integrity of the cells, Eosin–nigrosin staining was used to evaluated the viability of sperm according to the WHO protocol (15). Spermatozoa with red (D1) or dark pink (D2) heads are considered dead (membrane-damaged), whereas spermatozoa with white heads (L) or light pink heads are considered alive (membrane intact).

#### Diff-Quick staining 

Slides were stained with Diff–Quick staining. The dried colored slides were scanned at 100× magnification to look for morphological anomalies. A total of 200 spermatozoa per sample were classified according to their morphology; such as normal and abnormal heads, middle piece, and tail. The sum of the abnormal sperm was expressed as a percentage (16).

### Evaluation of sperm chromatin quality and apoptosis

#### Toluidine blue (TB) test

TB stain is a basic nuclear dye used for metachromatic and orthochromatic staining of chromatin. In fact, this stain is a sensitive structural probe for both sperm DNA structure and sperm chromatin packaging, because the test measures the accessibility of the sperm chromatin DNA phosphate residues for dye molecules, which is dependent on both the protein condition and DNA integrity (17, 18). In a study under an optical microscope using a magnification of 100 oculars, the quality of the chromatin of the spermatozoa was determined as a function of the metachromatic coloration of the heads of sperm with the following scores: score = 0 light blue (good chromatin); score 1 = dark blue (light abnormal chromatin); and score 2 = purple and violet (severe chromatin anomaly) (19). The total sperms with scores 1 and 2 were considered TB+ or abnormal chromatin while sperms scoring 0 as TB– or sperm with normal chromatin.

#### Aniline blue (AB) staining

AB staining was performed as previously described by Hofmann and Hilscher (20). AB staining is a kind of cytochemical test for detection of residual histones and therefore indirectly the presence of lower amounts of protamines in the sperm nucleus. (21). For each stained smear, 200 spermatozoa were evaluated with light microscope in oil immersion magnification (100x objective). Spermatozoa with unstained nuclei are considered normal (mature chromatin) while those blue stained were considered abnormal (immature chromatin).

#### TUNEL assay

Terminal deoxynucleotidyl transferase-mediated d-UTP nick end labeling assay (TUNEL) was first explained by Gorczyca et al. (1993) and used for direct detection of DNA fragmentation in mammalian spermatozoa. The principle of TUNEL test is labeling the 3'ends of fragmented DNA with biotinylated dUTP by means of recombinant terminal deoxynucleotidyl transferase (TdT) enzyme in a template independent manner. These incorporated labelled nucleotides can be distinguished in spermatozoa using flow cytometry, fluorescence microscopy and also light microscopy (22). The nuclei of spermatozoa with fragmented DNA (TUNEL+) showed a bright green color, the nuclei of normal cells (TUNEL–) were place green.

### Ethical considerations

All animal procedures were performed and approved in accordance with the guide for the laboratory animals' care and usage of the Medical University of Birjand, Iran (Ethic code: IR.BUMS.REC.1397.379). All efforts were made to minimize animal suffering and to reduce the number of animals used.

### Statistical analysis

Statistical analysis was performed using SPSS v. 22 (IBM, 2013). The results were presented as mean ± SD. A P-value < 0.05 was considered significant.

## 3. Results

**Figure 1:**
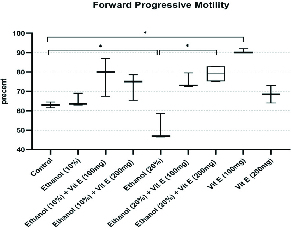
Comparison of the rate of forward progressive motility among the groups. Boxes depict the 25${}^{\mathrm{th}}$ and 75${}^{\mathrm{th}}$ percentiles with indication of the median and whiskers depict the 10${}^{\mathrm{th}}$ and 90${}^{\mathrm{th}}$ percentiles. *P < 0.05.

Figure 1 shows a comparison of the rates of forward progressive motility among the studied groups. Our findings revealed that 63% of spermatozoa retrieved from the cauda epididymis had forward progressive motility in the control group. This rate significantly decreased in group 5 (50.66%, p = 0.04). However, with respect to the group 5, a significant increase in the rate of forward progressive motility was seen in the group 7 (p = 0.04). Compared to the control group, a significant increase was observed in the forward progressive motility rate of the group 8 (p = 0.02).

**Figure 2:**
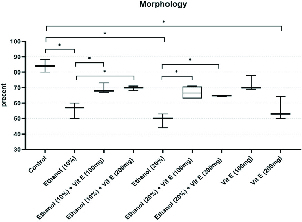
Comparison of the rate of normal morphology among the groups. Boxes depict the 25${}^{\mathrm{th}}$ and 75${}^{\mathrm{th}}$ percentiles with indication of the median and whiskers depict the 10${}^{\mathrm{th}}$ and 90${}^{\mathrm{th}}$ percentiles. *P < 0.05.

Figure 2 illustrates a comparison of the results of sperm normal morphology rate among the groups. Our results revealed that in the control group, 84% of the spermatozoa had normal morphology. The rate of normal morphology decreased significantly in the ethanol consuming groups 2 and 5 in comparison with the control group (p < 0.0001 and p < 0.0001, respectively). This rate increased significantly in ethanol and vit. E-consuming group. Interestingly, the rate of normal morphology in the vit. E 200 mg consumer group decreased significantly in comparison with the control group (p < 0.0001).

**Figure 3:**
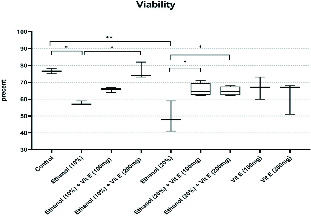
Comparison of the rate of sperm viability among the groups. Boxes depict the 25${}^{\mathrm{th}}$ and 75${}^{\mathrm{th}}$ percentiles with indication of the median and whiskers depict the 10${}^{\mathrm{th}}$ and 90${}^{\mathrm{th}}$ percentiles. *P < 0.05, **P < 0.01.

Figure 3 shows the results of the sperm viability rate. The sperm viability rate decreased significantly in the consuming groups 2 and 5 compared to the control group (p < 0.0001 and p < 0.0001, respectively). This rate was significantly increased in the ethanol and vit. E-consuming groups.

Table I presents the results of sperm chromatin staining and DNA integrity among the groups. For AB staining, the rates of AB-reacted spermatozoa were similar in all groups of animals except the group 9. The rate of spermatozoa with protamine deficiency was significantly increased in consumer group 9 than the control group (p = 0.01). Moreover, the results of the TB test showed that there was no significant difference between groups 2, 3 and 4 compared to the control group. However, the rates of spermatozoa with abnormal DNA structure and packaging increased in the ethanol consumer group 5 with respect to the control group (p = 0.01). Although, the difference between groups 5 and 6 was not significant, a significant decrease in the rate of TB-reacted spermatozoa in the group 7 with respect to the group 5 was seen (p = 0.007). The result of the TUNEL test showed that the rate of DNA fragmentation was significantly increase in the ethanol group 5 than the control group (p = 0.04). Furthermore, we observed a significant decrease in the rate of DNA fragmentation in treated groups 6 and 7 with respect to the consuming group 5 (p ≤ 0.001 and p ≤ 0.001, respectively). The rate of TUNEL+ spermatozoa did not show a significant difference between the -treated groups 2, 7 and 8 compared to the control group.

**Table 1:** Percentage of AB+, TB+, and TUNEL+ spermatozoa in the experimental groups


	**Group 1: Control**	**Group 2: Ethanol (10%)**	**Group 3: Ethanol (10%) + Vit. E (100 mg)**	**Group 4: Ethanol (10%) + Vit. E (200 mg)**	**Group 5: Ethanol (20%)**	**Group 6: Ethanol (20%) + Vit. E (100 mg)**	**Group 7: Ethanol (20%) + Vit. E (200 mg)**	**Group 8: Vit. E (100 mg)**	**Group 9: Vit. E (200 mg)**
**AB+**	6.5 ± 0.70	6.33 ± 0.57	7.66 ± 1.15	8 ± 1.732	6 ± 1	7.66 ± 0.57	7.25 ± 0.95	9.33 ± 1.52	10.33 ± 0.57${}^{a}$
**TB+**	7 ± 1	7 ± 0	6.66 ± 1.15	8 ± 2	11.66 ± 2.51${}^{b}$	10.66 ± 1.52	7 ± 1${}^{c}$	7.33 ± 1.52	6.66 ± 1.15
**TUNEL+**	6.33 ± 1.15	7 ± 1	4 ± 1	4 ± 1	9.66 ± 0.57${}^{d}$	4.75 ± 0.95${}^{e}$	5.5 ± 1.29${}^{f}$	5.66 ± 1.52	5.33 ± 1.52
Data presented as Mean ± SD. ${}^{a}$Difference between vit. E 200 mg and control group (p = 0.0163), ${}^{b}$Difference between ethanol 20% and control group (p = 0.0142), ${}^{c}$Difference between ethanol 20% + vit. E 200 mg and ethanol 20% group (p = 0.0077), ${}^{d}$Difference between ethanol 20% and control group (p = 0.0455), ${}^{e}$Difference between ethanol 20% + vit. E 100 mg and ethanol 20% group (p = 0.0005), ${}^{f}$Difference between ethanol 20% + vit. E 200 mg and ethanol 20% group (p = 0.0032), AB: Aniline blue staining, TB: Toluidine blue test, TUNEL: Terminal deoxynucleotidyl transferase dUTP nick end labeling									

## 4. Discussion

However, to the best of our knowledge, up to now, the probable inhibitory effects of vit. E on the adverse effects of alcohol consumption have not been studied. In this study, the possible protective effects of Vit. E on the alterations of alcohol intake on different sperm parameters including motility, abnormal morphology, viability, and changes of chromatin in the animal model (BALB/c mice) were investigated. Our findings showed that sperm forward progressive motility, normal morphology rate, and viability decreased significantly in ethanol 10%– and 20%–treated groups when compared with the control group. While cotreatment with vit. E could prevent some of these adverse effects, studies have reported the adverse effects of alcohol abuse on semen parameters (23, 24). One of the mechanisms associated with alcohol consumption is its direct effect on testosterone metabolism and spermatogenesis. With alcohol consumption, the ratio between estradiol and testosterone is disturbed, causing spermatogenic arrest and the Sertoli cell only syndrome. Following alcohol consumption, it interferes with the function of the hypothalamic–pituitary–testicular axis, impairs gonadotropin secretion, and lowers testosterone levels (25).

Oxidative stress is one of the most important factors that causes the loss of sperm motility. One of the main cellular sources of ROS in semen is sperm. Male germ cells are able to produce small amounts of ROS, such as superoxide anion, hydrogen peroxide, and nitric oxide, which interfere with sperm chromatin density if the ROS increases (26).

In normal sperm, ROS plays an important role in capacity, acrosome response, mitochondrial function, and sperm motility. ROS can also play a significant role in cellular activity as a secondary messenger (27); “Vit. E, also known as alpha-tocopherol, is a fat-soluble antioxidant vitamin found in almonds, avocados, spinach, and sweet potatoes that neutralizes free radicals and protects the cellular membrane against damage from ROS. It prevents lipid peroxidation and enhances the function of other antioxidants. The lipophilic character of vit. E enables it to locate itself in the interior of the cell membrane bilayer, where it rapidly reacts with fatty acid peroxyl radicals, the primary products of lipid peroxidation, and intercepts the chain reaction" (10).

Joo and colleagues reported that alcohol consumption led to a significant increase in abnormal morphological nuclei and plasma membranes (28). In line with our findings, Rahimipour and coworker reported that the rate of abnormal morphology was increased in mice treated with ethanol (29). While the morphology was not decreased significantly in the group receiving vit. E 100 mg compared to the control group, it decreased significantly in the group receiving vit E 200 mg compared to the control group, however, we couldn't find an explanation for this decline.

Hence, it seems alcohol can reduce sperm motility by decreasing sperm normal morphology rate. In another study, the epididymal sperm motility was shown to decrease in the ethanol-consuming group (29). Similarly, in the current study, alcohol intake decreased sperm normal morphology and progressive motility. Considering that this increase in motility was not significant in the ethanol 10% group compared to the control group, it seems that the antioxidant system of the mouse body was able to prevent the effect of ethanol 10% on sperm motility, but in the ethanol 20% group decrease in motility was significant, which may indicate the threshold dependent manner effect of ethanol on sperm motility.

OS is detrimental to cell function like sperm motility and many hypotheses have been proposed to explain the association between OS and decreased sperm motility (30, 31). H${}_{2}$O${}_{2}$ has been determined to induce lipid peroxidation, and not only disrupts sperm motility but also impairs all of the functions of sperm that depend on the integrity of cell membranes (32). Disruption of the mitochondrial membrane leads to reducing its ATP content results in decreasing sperm motility (33). There are many reports that alcohol consumption can increase ROS through a variety of pathways (34, 35). One of the possible mechanisms of reducing sperm motility in this study can be related to the ROS elevation level in the alcohol-consuming groups.

In line with our findings, Rahimipour and colleagues reported that ethanol could decrease sperm vitality in mice treated with ethanol (36). Also, the results of our study in this regard were consistent with the results of Poor Entezari's and their colleagues' (37). Viability was not decreased significantly in the group receiving vit. E 100 and 200 mg compared to the control group, and we couldn't find an explanation for this slight decline.

Thus, in the present investigation, we observed that a coadministration of vit. E with ethanol could repair the ethanol-induced impairments of sperm forward progressive motility, normal morphology rate, and viability compared to the ethanol-consuming groups. There is evidence related to significant roles of some vitamins on the maintenance as well as regulating functions in the male reproductive system which is attributed to their antioxidant properties (11). Studies have shown useful effects of the administration of vit. E on sperm functions in animals (13, 14).

Biswas and coworker have reported that the intake of vit. E protects the spermatozoa by maintaining the cell membranes fluidity by stabilizing the polyunsaturated fatty acids (38). Ourique et al. reported that the administration of vit. E may increase total cellular levels of free radical scavenging activity through enhancement of antioxidant capacity on testes and epididymides in rats (14).

In the process of spermiogenesis, the nuclei of the spermatids are conditioned, and in this phenomenon, the histones are replaced by nuclear proteins specific to the testes, called transition proteins and finally by the other proteins called protamins. These step-by-step replacements increase chromatin compactions. It is believed that this type of nuclear chromatin architecture protects the sperm genome from damage caused by stressful conditions such as OS, temperature, and denaturation induced by DNA during transport in the female reproductive system (39). “There are three main causes of sperm DNA damage including abnormal chromatin packaging during spermiogenesis, abortive apoptosis, and excessive production of ROS” (36).

AB staining shows sperm cells with excessive histones and incomplete protamine replacement. This staining in our study revealed no significant difference between the groups consuming ethanol compared to the control group. This finding is consistent with that of Talbi and coworker who showed that the levels of sperm reacted with AB were similar in the ethanol-consuming groups and in the control group. They concluded that alcohol consumption did not increase the level of sperm with residual histones (40). However, our result contrasts with previous studies which have shown that alcohol consumption in mice has harmful effects on the replacement of histones-protamines during the compacting of sperm chromatin (36). This finding is consistent with that of Pourentezari et al. study (37) who showed an increase in excessive histones and incomplete protamine replacement in sperm with alcohol-treated mice compared to controls.

TB staining is a good test to check for defective DNA structure and packaging. According to TB staining, we found a significant difference between the groups consuming alcohol compared to the control group. Pourentezari and coworker (37), showed that TB staining indicates a significant difference between alcohol groups compared to control group.

In addition, a significant difference between the groups treated with alcohol and vit. E compared to the groups consuming alcohol was observed. This finding implies that vit. E is able to counteract the ethanol-induced impairment of sperm abnormal DNA structure and packaging. Our findings of this parameter are similar to the results of previous studies in which the rate of TB-positive spermatozoa had been increased in the alcohol-consumption group compared to the control group (36, 41).

Zhu and colleagues showed that in alcoholic men, the rate of apoptosis had increased in germ cells (41). This finding had been assessed based on the fragmentation of sperm DNA by using the TUNEL assay (39). The TUNEL assay is also used to detect DNA strand breakage which is the major sign of sperm apoptosis (42). Our findings in the current study demonstrated that significant differences were found for chromatin damages in ethanol-consumer groups. One possible mechanism for increasing sperm nuclear DNA damage in alcohol-consuming mice is OS. ROS is the major source of DNA damage, strand breakage, and a variety of alterations in the nucleotides (43).

Although in the present study we did not evaluate the ROS level, there are several reports indicating that alcohol consumption can elevate the ROS level (44) such as the results of Rahimipour and colleagues (36), which are in line with us. These researches showed that ethanol consumption disturbs the DNA integrity of spermatozoa in mice. Finally, we recommend the administration of vit. E to prevent or neutralize the undesirable effects of ethanol in alcoholics.

## 5. Conclusion

The findings of this study showed that sperm forward progressive motility, normal morphology rate, and viability decreased significantly in ethanol-treated groups; also, the rates of spermatozoa with abnormal DNA structure and DNA fragmentation increased significantly in the ethanol-taking groups than the control group. While a cotreatment with vit. E could prevent some of these adverse effects, it can be concluded that vit. E is able to counteract the ethanol-induced impairment of sperm parameters, abnormal DNA structure, packaging, and fragmentation.

##  Conflict of Interest

The authors declare that there is no conflict of interest.

## References

[B1] Flyckt R, Falcone T (2019). Infertility: A practical framework. Cleve Clin J Med.

[B2] Agarwal A, Mulgund A, Hamada A, Chyatte MR (2015). A unique view on male infertility around the globe. Reprod Biol Endocrinol.

[B3] La Vignera S, Condorelli RA, Balercia G, Vicari E, Calogero AE (2013). Does alcohol have any effect on male reproductive function? A review of literature. Asian J Androl.

[B4] Muthusami KR, Chinnaswamy P (2005). Effect of chronic alcoholism on male fertility hormones and semen quality. Fertil Steril.

[B5] Bisht Sh, Faiq M, Tolahunase M, Dada R (2017). Oxidative stress and male infertility. Nat Rev Urol.

[B6] Gavriliouk D, Aitken RJ (2015). Damage to sperm DNA mediated by reactive oxygen species: Its impact on human reproduction and the health trajectory of offspring. Adv Exp Med Biol.

[B7] Doostabadi M, Afshar M, Hosseini M, Ezi S, Hassanzadetaheri M (2016). Protective effect of aqueous jujube extract in Carbamazepine induced teratogenicity on Balb/c mice fetuses. IJABR.

[B8] Bhardwaj JK, Mitta M, Saraf P, Kumari P (2020). Pesticides induced oxidative stress and female infertility: A review. Toxin Rev.

[B9] Ricci E, Noli S, Ferrari S, La Vecchia I, Cipriani S, De Cosmi V, et al (2018). Alcohol intake and semen variables: Cross-sectional analysis of a prospective cohort study of men referring to an Italian Fertility Clinic. Andrology.

[B10] Zwolak I (2020). Protective effects of dietary antioxidants against vanadium-induced toxicity: A review. Oxid Med Cell Longev.

[B11] Majzoub A, Agarwal A (2018). Systematic review of antioxidant types and doses in male infertility: Benefits on semen parameters, advanced sperm function, assisted reproduction and live-birth rate. Arab J Urol.

[B12] Asadi N, Bahmani M, Kheradmand A, Rafieian-Kopaei M

[B13] Zubair M (2017). Effects of dietary vitamin E on male reproductive system. Asian Pac J Reprod.

[B14] Ourique GM, Saccol EMH, Pes TS, Glanzner WG, Schiefelbein SH, Woehl VM, et al (2016). Protective effect of vitamin E on sperm motility and oxidative stress in valproic acid treated rats. Food Chem Toxicol.

[B15] World Health Organization (2010). WHO laboratory manual for the examination and processing of human semen.

[B16] Kishikawa H, Tateno H, Yanagimachi R (1999). Chromosome analysis of BALB/c mouse spermatozoa with normal and abnormal head morphology. Biol Reprod.

[B17] Talebi AR (2011). Sperm nuclear maturation: A basic and clinical approach.

[B18] Agarwal A, Sharma R, Ahmad G, Gardner DK, Weissman A, Howles CM, Shoham Z, editors (2017). Sperm chromatin assessment. Textbook of assisted reproductive techniques. 5 th Ed.

[B19] Tsarev I, Bungum M, Giwercman A, Erenpreisa J, Ebessen T, Ernst E, et al (2009). Evaluation of male fertility potential by Toluidine Blue test for sperm chromatin structure assessment. Hum Reprod.

[B20] Hofmann N, Hilscher B (1991). Use of aniline blue to assess chromatin condensation in morphologically normal spermatozoa in normal and infertile men. Hum Reprod.

[B21] Kazerooni T, Asadi N, Jadid L, Kazerooni M, Ghandi AR, Ghaffarpasand F, et al (2009). Evaluation of sperm’s chromatin quality with acridine orange test, chromomycin A3 and aniline blue staining in couples with unexplained recurrent abortion. J Assist Reprod Genet.

[B22] Evenson DP (2016). The sperm chromatin structure assay (SCSA) and other sperm DNA fragmentation tests for evaluation of sperm nuclear DNA integrity as related to fertility. Anim Reprod Sci.

[B23] Maneesh M, Dutta S, Chakrabarti A, Vasudevan DM (2006). Alcohol abuse-duration dependent decrease in plasma testosterone and antioxidants in males. Indian J Physion Pharmacol.

[B24] Nagy F, Pendergrass B, Bowen DC, Yeager JC (1986). A comparative study of cytological and physiological parameters of semen obtained from alcoholics and non-alcoholics. Alcohol Alcohol.

[B25] Ricci E, Al Beitawi S, Cipriani S, Candiani M, Chiaffarino F, Vigano P, et al (2017). Semen quality and alcohol intake: A systematic review and meta-analysis. Reprod BioMed Online.

[B26] Agarwal A, Sekhon LH (2010). The role of antioxidant therapy in the treatment of male infertility. Hum Fertil.

[B27] Sheweita SA, Tilmisany AM, Al-Sawaf H (2005). Mechanisms of male infertility: Role of antioxidants. Curr Drug Metab.

[B28] Joo KJ, Kwom YW, Myung SC, Kim TH (2012). The effects of smoking and alcohol intake on sperm quality: Light and transmission electron microscopy findings. J Int Med Res.

[B29] Martinez M, Macera S, de Assis GF, Pinherio PFF, Almeida CCD, Tirapelli LF, et al (2009). Structural evaluation of the effects of chronic ethanol ingestion on the testis of Calomys callosus. Tissue Cell.

[B30] Armstrong JS, Rajasekaran M, Chamulitrat W, Gatti P, Hellstrom WJ, Sikka SC (1999). Characterization of reactive oxygen species induced effects on human spermatozoa movement and energy metabolism. Free Radic Biol Med.

[B31] Makker K, Agarwal A, Sharma R (2009). Oxidative stress and male infertility. Indian J Med Res.

[B32] Bansal AK, Bilaspuri GS (2010). Impacts of oxidative stress and antioxidants on semen functions. Vet Med Int.

[B33] Dias TR, Alves MG, Silva BM, Oliveira PF (2014). Sperm glucose transport and metabolism in diabetic individuals. Mol Cell Endocrinol.

[B34] Zakhari S

[B35] Wu D, Cederbaum AI (2003). Alcohol, oxidative stress, and free radical damage. Alcohol Res Health.

[B36] Rahimipour M, Talebi AR, Anvari M, Abbasi Sarcheshmeh A, Omidi M (2013). Effects of different doses of ethanol on sperm parameters, chromatin structure and apoptosis in adult mice. Eur J Obstet Gynecol Reprod Biol.

[B37] Pourentezari M, Talebi AR, Mangoli E, Anvari M, Rahimipour M (2016). Additional deleterious effects of alcohol consumption on sperm parameters and DNA integrity in diabetic mice. Andrologia.

[B38] Biswas A, Mohan J, Sastry KVH (2009). Effect of higher dietary vitamin E concentrations on physical and biochemical characteristics of semen in Kadaknath cockerels. Br Polut Sci.

[B39] Carrell DT, Emery BR, Hammoud S

[B40] Talebi AR, Abbasi Sarcheshmeh A, Khalili MA, Tabibnejad N (2011). Effects of ethanol consumption on chromatin condensation and DNA integrity of epididymal spermatozoa in rat. Alcohol.

[B41] Zhu Q, Meisinger J, Emanuele MA, La Paglia N, Van Thiel DH (2000). Ethanol exposure enhances apoptosis within the testes. Alcohol Clin Exp Res.

[B42] Tavalaee M, Nasr-Esfahani MH, Deemeh MR (2008). Etiology and evaluation of sperm chromatin anomalies. Int J Fertil Steril.

[B43] Cooke MS, Evans MD, Dizdaroglu M, Lunec J (2003). Oxidative DNA damage: Mechanisms, mutation, and disease. FASEB J.

[B44] Bailey SM, Patel VB, Young TA, Asayama K, Cunningham CC (2001). Chronic ethanol consumption alters the glutathione/glutathione peroxidase-1 system and protein oxidation status in rat liver. Alcohol Clin Exp Res.

